# Mechanisms of enhancer action: the known and the unknown

**DOI:** 10.1186/s13059-021-02322-1

**Published:** 2021-04-15

**Authors:** Anil Panigrahi, Bert W. O’Malley

**Affiliations:** Department of Molecular and Cellular Biology, Baylor College of Medicine, One Baylor Plaza, Houston, TX 77030 USA

## Abstract

Differential gene expression mechanisms ensure cellular differentiation and plasticity to shape ontogenetic and phylogenetic diversity of cell types. A key regulator of differential gene expression programs are the enhancers, the gene-distal *cis*-regulatory sequences that govern spatiotemporal and quantitative expression dynamics of target genes. Enhancers are widely believed to physically contact the target promoters to effect transcriptional activation. However, our understanding of the full complement of regulatory proteins and the definitive mechanics of enhancer action is incomplete. Here, we review recent findings to present some emerging concepts on enhancer action and also outline a set of outstanding questions.

**Supplementary Information:**

The online version contains supplementary material available at 10.1186/s13059-021-02322-1.

## Introduction

Metazoans—the multicellular organisms that comprise the Kingdom Animalia—began their formative journey of evolution over 635 million years ago, when multicellularity emerged from early unicellular holozoans [1, 2]. Multicellularity conferred increased size, greater metabolic competence, protection from predation, and division of labor through functional diversification and specialization of cells, ensuring increased chance of survival and evolutive fitness [3]. The human body is composed of over 37 trillion cells [4] that make up hundreds of different cell types despite sharing identical genotype [5]. Such cellular diversity and morphological complexity have been achieved through cellular differentiation, which integrates both evolution and development [6, 7]. In addition to differentiation, cells are constantly challenged by various physicochemical assaults and thus must develop phenotypic plasticity [8]. At the heart of cellular plasticity and differentiation is the drive to acquire proteins with new functions or to alter their spatiotemporal patterns of expression. This necessitates making new proteins, or repurposing existing proteins for novel functions. Such diversity in protein expression during evolution is acquired through gaining or losing genes, as well as by acquiring differential gene regulatory capabilities [9, 10]. Cells achieve differential gene expression by implementing complex gene regulatory networks [11, 12] that may include many alternate layers of non-transcriptional gene regulation [13]. A gene regulatory network can be described as complex interplays between two components: the target gene and its regulators. The regulators consist of both the *cis-*regulatory elements (CRE) in the genome and relevant gene products; the latter includes proteins (as *trans*-acting transcription factors (TF) and signaling molecules) and regulatory non-coding transcripts. The CRE repertoire includes the boundary elements or insulators necessary for specific spatial organization of the chromatin into topologically associated domains (TAD), the silencers, the enhancers (distal sequence elements that activate a target gene’s expression), and the promoters. Experimental evidence strongly suggests that the distant enhancers physically contact the promoters by chromatin looping during transcription activation. However, how exactly the enhancer accomplishes the over-all act of gene activation is still unresolved—even nearly 40 years after its discovery as a 72 bp repeat element in the Simian Virus 40 genome [14–16]. In this review, we attempt to summarize our most current understanding of enhancer function and to outline the questions yet to be resolved. Different aspects of this review are subjects of several excellent reviews with varied coverage [17–21].

## Enhancers

### General features of enhancers

Enhancers are non-coding sequences in the genome that activate the expression of target genes transcribed by the RNA polymerase II (RNAPII). Enhancers can act independent of orientation, distance, and location with respect to the target gene [14] and can be located over as much as a million base pairs away—as seen for the *SHH* gene [22]. Readers are referred to an excellent recent review that details the development of the concept of transcriptional enhancers [23]. Vertebrate enhancers can be 100–1000 bp in length, and multiple enhancers can exist in a cluster to form a super-enhancer [24]—analogous to previously described locus control regions [25]. Enhancers are found mostly in the intergenic and intronic regions, while a few enhancers have been found within exons. As detailed below, enhancers consist of dense clusters of transcription factor binding sites (TFBS) and are bound by cell type-specific TFs, coregulators, chromatin modifiers, architectural proteins like Cohesin, Condensin and CTCF, other enzymes, and RNAPII. Owing to such large-scale protein assembly, enhancers are often nucleosome deficient, and thus are hypersensitive to nucleases reflecting DNA accessibility—a feature widely exploited as a signature of enhancers [26]. Thus assembled, the enhancer complex loops over and physically contacts the target promoter and activates transcription.

### Origin and evolution of metazoan enhancers

Several lines of reasoning argue that the early holozoan relatives of the metazoans already possessed enhancer-like regulatory features. Enhancer-like elements exist in bacteria that regulate target gene expression by DNA looping [27, 28]. The upstream activating sequences (UAS) are prevalent in yeasts and also can function as enhancers in human cells [29]. The plants have also evolved transcriptional enhancers [30], even as the ancestors of plants and animals diverged very early in eukaryotic evolution nearly two billion years ago [31, 32]. Moreover, microsynteny is evidently conserved across metazoans [33, 34], indicative of the presence of enhancers in the bystander genes. Also, comparative genomics has revealed a core group of paneukaryotic TFs [35]. Extensive functional genomic studies in an early pre-metazoan *Capsaspora owczarzaki* have identified dynamic cis-regulatory landscapes [36]. Among the early metazoans, enhancers were also identified in the sponge *Amphimedon queenslandica* [37] that can activate genes in zebrafish and mouse tissues [38]. Early metazoans have complex cellular differentiation patterns indicative of well-structured gene regulatory mechanisms [39, 40]. These findings collectively establish that enhancers and enhancer-like gene regulatory mechanisms predate metazoan evolution. Being nucleosome-deficient, enhancers exhibit higher mutation rate [26], suggesting that the pre-metazoan cis-regulatory regions could have been the ideal breeding grounds for enhancer evolution. Indeed, it is now recognized that new cis-regulatory motifs emerge from pre-existing regulatory sequences through co-option or exaptation [41–43]. Additionally, enhancers also emerge from transposable elements (TE) [44, 45]; nearly half of the human genome consists of TEs [46]. Also, the inherent transposition process introduces terminal duplication and sequence diversity, making the TEs ideal tools to cause widespread changes to the genomic landscape. Unsurprisingly, both the DNA transposons and retrotransposons have been widely reported as the source of enhancer activity [47–51].

Genomic duplication events—be it tandem duplications of chromosomal regions, duplications of whole chromosomes, or whole genomes—also create enhancers. One of the copies post duplication is essentially redundant and can tolerate mutations, while the genetic integrity of the other copy is maintained. This results in complete or partial loss of the duplicated region (non-functionalization or sub-functionalization), or gain of new functional features (neo-functionalization). If the duplicated region was a cis-regulatory module, it evolves new regulatory functions over time, resulting in the genesis of new enhancers [52, 53]. In a related scenario, when a CRE-coupled-gene module is duplicated, both the resultant CREs develop specializations such that the paralogs are regulated differently [54, 55]. Additionally, TFBSs can be created de novo in an inert sequence, which can evolve over time into an enhancer. Computational simulations predict that a 6 bp TFBS can emerge in *Drosophila* in 24 years [56], while a complex CRE with multiple TFBSs can take over 0.5 million years to emerge [57]. Some enhancers reportedly emerged de novo from ancestral or extant exons [58–60]; however, there is no evidence of the emergence of any enhancer in a region that did not have any ancestral history of transcription [61]. Prior transcriptional status supports enhancer emergence; because high indels, substitutions and recombination are conducive to enhancer evolution, and correlate very strongly with open chromatin in germline genomes [62]. Nevertheless, protein-coding sequences have diverged less compared to the non-coding sequences. Consistent with this fact, it is accepted that the emergence and subsequent divergence of CREs, but not the protein-coding sequences per se, led to the evolution of morphological phenotypes [6], a notion initially proposed more than 55 years ago [63].

### Sequence features of enhancers

Enhancers are evolutionarily conserved in sequence and function [64]. For example, thousands of highly conserved non-coding sequences representing potential enhancers are found in all jawed vertebrates [65]. However, enhancers may display sequence divergence despite exhibiting conserved function [66, 67]. Enhancer function is a reflection of its underlying sequence, which largely contains dense clusters of TFBSs [68]. The functional potential of an enhancer is influenced by several parameters including the type of the TF encoded to bind the enhancer sequence, the orientation, binding affinities, order, number, and spacing of individual TFBSs along the enhancer, and the underlying DNA topology, collectively called “enhancer architecture” [69–71]. It is believed that the inactive enhancers normally exist in closed chromatin conformation because the DNA sequence underlying CREs has a high potential to form nucleosomes [72]. However, enhancers undergo rapid nucleosome depletion upon TF binding [73].

Two questions arise here: why do enhancers contain so many TFBSs, and why are some ultra-conserved in evolution while others are not? From the perspective of chromatin packaging, it is useful to cluster multiple TFBSs together, so that additive binding of the required TFs could progressively overcome the higher propensity of the enhancers to form nucleosomes, allowing desired gene regulation. From the perspective of cellular phenotypic diversity, a given enhancer can regulate a cognate gene in multiple cell types differentially or temporally if they express multiple different TFs that bind the enhancer. Thus, availability of TFBSs allows the different cell types to employ their specific TFs to utilize the same enhancer and yet choose the timing and magnitude of its action. The major types of enhancer architecture are the “billboard,” “TF collective,” and the “enhanceosome” models [68, 70]. The TFBSs in many enhancers are modular in nature. That is, a few neighboring TFBSs form a module and almost function independently of the rest of the TFBSs in the enhancer as the TFs bind additively [74]. This enhancer architecture confers low conservation in TFBS sequences and supports additive or cooperative TF recruitment, known as a “billboard model.” In the “TF collective” model, TFs can be recruited through their respective TFBSs as well as through protein-protein interactions. Since certain TFs can bind the enhancer indirectly, these enhancers are expected to display higher evolvability, and thus exhibit lower conservation. It is possible for such enhancers to display similar TF occupancy despite having diverged sequence motifs, and dissimilar TF occupancy despite conserved sequences [75]. On the contrary, the “enhanceosome” enhancers, such as the one regulating the human *IFNB1* gene, contain multiple interdependent domains within, such that alterations in the sequence, order, or spacing of the domains are not tolerated, and the TFs bind cooperatively as one functional unit [76]. These enhancers represent a class that is under high evolutive constraints and thus highly conserved in evolution.

### Proteome of enhancers

**Fig. 1:**
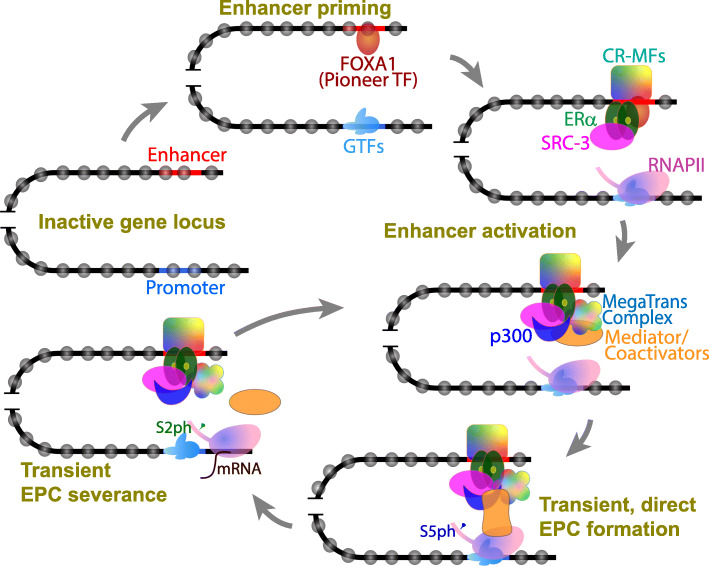
A simplistic schematic for enhancer-mediated transcription activation. A pioneer TF binds a nucleosome at an enhancer and nucleates the process of enhancer priming, facilitating the recruitment of other TFs (ERα) and chromatin remodeling and modification factors (CR-MFs). ERα recruits the coactivators SRC-3 and p300, plus other TFs and coactivators as the MegaTrans complex. The Mediator complex and other relevant coactivators with distinct enzyme activities are subsequently recruited and their conformational changes result in transfer of the enhancer-bound coactivators onto the promoter-bound RNAPII complexes, establishing “transient and direct” enhancer-promoter contact (EPC). Sequential phosphorylation of the RNAPII C-terminal domain (CTD) at S5 and S2 residues coordinates transcription initiation and transition to elongation. Once the RNAPII exits the promoter for productive transcript elongation (mRNA), the Mediator/coactivators can enter another round of recruitment to the enhancer and subsequent transfer to the promoter, aided by EPC formation and severance, completing another cycle of RICE (collective “RNAPII/coactivator ***r***ecruitment ➔ transcription ***i***nitiation➔ promoter ***c***learance ➔productive ***e***longation” events). Similar actions by other TFs at the enhancer can amplify TF action, resulting in synergistic transcription activation. Not shown for simplicity: GTFs, RNAPII and eRNA transcription at the enhancer, and other factors and CR-MFs at the promoter

During a lineage-specification event, pioneer TFs access their nucleosomal binding sites at “closed” enhancers [77]. This pioneering event is followed by cooperative binding of other TFs to their cognate binding sites freed of nucleosomes, aided by chromatin remodeling factors and histone acetyl transferases (HAT) [78]. What follows, it is believed, is sequential or collective recruitment of other TFs, coregulators, chromatin remodelers and modifiers, and RNAPII (Fig. 1 [79]). Enhancer priming and activation likely occurs in successive phases of protein recruitment that may involve assisted loading of additional TFs [80] and coregulator exchange [81]. Thus, enhancer activation results in a large assembly of hundreds of proteins [82–84]. Naturally, such large-scale protein recruitment keeps the enhancer region nucleosome deficient and thus hypersensitive to nucleases [85, 86], a feature popularly utilized to identify enhancers [26]. Proteins assembled at enhancers can promote either activation or repression of target genes depending on their repertoire of coactivator or repressor complexes, respectively [87, 88].

The proteome at the enhancer can be broadly classified into five groups. These include TFs, architectural proteins like the histones, coactivators that are recruited to the enhancers via TFs, reader proteins that recognize specific modifications on the underlying histones, and finally RNAPII and other enzymes that catalyze or influence various steps to ensure stepwise transition of transcription from formation of the pre-initiation complex (PIC) to productive transcription through promoter clearance, elongation, proximal pausing, and pause release (see Table 1).

**Table 1:** Proteome of enhancers

Protein categories	Select examples	Select references
TFs	Sequence-specific TFs that bind enhancers directly and other TFs recruited via protein-protein interactions—classified into distinct structural and functional groups	[68, 82, 83, 89, 90]
	General Transcription Factors (GTFs)	[91–93]
Architectural proteins	Chromatin enriched with histone H3K4me1, H3K27ac; H3.3, H2Az	[94]
	Cohesin, Condensin, CTCF	[82, 95–97]
Coregulators	Mediator complex	[95, 98]
	Chromatin remodeling complexes and chromatin-modifying enzymes (“writers” and “erasers” of post-translational modifications)	[99]
	Integrator complex	[100]
	Steroid receptor coactivators and other coregulators of TFs	[81, 83, 84]
Effector proteins	Proteins that “read” histone post-translational modifications	[94, 101]
Catalytic enzymes	RNAPII and associated enzymes	[93]
	Other enzymes (RNA exosome complex; PARP-1; Topoisomerase-1; TET2, etc.)	[102–105]

The enhancer itself undergoes transcription (discussed below) and is enriched with the RNAPII and all the general transcription factors (GTFs) [91, 92]. Not only the enhancers and promoters share similar sequence architecture and chromatin modifications, but also the promoters can function as enhancers [106]. A typical PIC may comprise the RNAPII and GTFs, totaling up to a sum of 45 proteins with a combined mass of over 2500 kDa [107]. Adding to that all the other groups of proteins mentioned above (Table 1), a modest estimate tells us that an active enhancer proteome may include up to two hundred proteins—excluding the promoter proteome in contact. It is inconceivable that each of these proteins has an indispensable role at the enhancer or at one connecting promoter; nevertheless, most of these proteins must cooperatively execute a series of interactions and biochemical reactions that culminates in the activation of the target promoter. We have only just begun to understand the functions of some of these players. For example, despite the activities of chromatin remodelers [108] and modifiers [109] having been elucidated extensively, we do not comprehensively know what substrates these activities act upon at the enhancer-promoter contacts (EPC) in what precise kinetics and to what precise impact. A systematic investigation to comprehensively identify and characterize all enzyme activities at the enhancers and promoters is warranted.

### Enhancers as the command centers for signaling pathways

Living systems are surrounded by and are exposed to a wide range of environmental factors. For example, the human exposome is comprised of thousands of biotic agents and chemical compounds [110]. One way these agents impact living systems is by instigating differential gene regulation, indicating that gene-regulatory instructions must be transduced from these external agents to the genome. The exposome constituents interact with specific receptor molecules on the cell surface that initiate cascades of diverse signaling pathways. The end result of the signaling processes is activation of specific TFs that bind to their cognate TFBSs at the CREs [111]. Such TF recruitment is essentially diverse and combinatorial, which ultimately dictates which gene is to express when and in what quantity. Signal transduction pathways employ over 1600 TFs to effectuate specific gene expression programs [90, 112]. The vast majority of enhancers respond to signaling cues and accordingly execute the requisite transcriptional programs. Thus, enhancers function as the command centers for signaling pathways. While external transcriptional stimuli (such as hormone induction) and differentiation can nucleate priming and activation of enhancers as discussed above, the active state of certain enhancers may be maintained in terminally differentiated cells to ensure sustained expression of housekeeping genes [113]. Thus, enhancers can be either inducible or constitutive.

Steroid hormone signaling presents a well-studied example of enhancer-mediated transcription regulation [114, 115]. The nuclear hormone receptors (NRs) such as the progesterone receptor (PR), androgen receptor (AR), glucocorticoid receptor (GR), and estrogen receptor (ER) are known to dimerize upon ligand binding while interacting with the target nuclear hormone response elements (NREs) at the enhancers, first demonstrated for GR and PR [116]. While some NRs can bind the NRE within a nucleosome in vitro, the stability and efficiency of such binding requires additional TFs [117, 118]. Inside the cells, the NRs invariably bind nuclease-accessible regulatory elements [119] and colocalize with a pioneer TF such as FOXA1 [120], though ER and GR can facilitate the recruitment of each other [121]. The NRs and the pioneer TFs often cooperate to regulate their mutual genomic occupancy [122]. Once bound to DNA, NRs promote both formation and stabilization of the PIC [123, 124]. The NRs usually recruit one of the three major steroid receptor coactivators (SRCs 1, 2, or 3) belonging to the p160 superfamily [87, 125]. Structural studies with cryo-electron microscopy have suggested that ERα and AR recruit SRC-3 and also bring in secondary coactivators to modify the chromatin neighborhood [126, 127]. ERα also mediates establishment of transcription-conducive chromatin interaction landscapes involving the enhancer, promoter, and gene body [128, 129], likely with the aid in part of the assembly of dynamic coregulator complexes [84]. Not surprisingly, impairment in hormone signaling and the consequent transcriptional dysregulation are implicated in diseases such as cancer [130].

### RNAs at enhancers

Apart from the DNA sequence motifs and assembled proteins, non-coding RNAs comprise another integral component of enhancers [131]. Although intergenic transcription had been observed at the β-globin loci much earlier [132, 133], widespread intergenic transcription was detected at genomic scale in 2005  [134, 135]. A bulk of these transcripts were long non-coding RNAs, while the rest mapped to enhancers [136, 137]. These transcripts are today widely known as enhancer-derived RNA, or eRNA. Studies employing CAGE (cap analysis of gene expression) have suggested that tens of thousands of distinct eRNAs can be detected in vertebrate cells, which outnumber the mRNAs [138, 139]. eRNAs can be bidirectional, divergent transcripts originating at about 180 bp apart, can be up to 2 kb long, largely unspliced and non-polyadenylated [19, 140, 141]. Although bidirectional transcription is supposedly a general feature of accessible chromatin not confined to enhancers [142], a recent single-cell transcriptome analysis argues that eRNAs are invariably unidirectional and non-divergent [143]. Depending upon the model system, methods of detection, and depth of analysis, eRNA synthesis may seem to precede promoter activation or can occur in concert with promoter-driven transcription. For example, lipopolysaccharide (LPS)-induced eRNA synthesis in mouse macrophages appears to precede transcription from the target promoter [136], whereas synchronous, E2-inducible eRNA and mRNA synthesis is observed in MCF-7 cells as assayed by global run-on sequencing (GRO-seq) [144]. CAGE analysis encompassing 33 time-course studies of cellular differentiation or activation has revealed a high degree of co-occurrence of transcription at both enhancers and promoters in both human and mouse cells [139]. eRNAs are short-lived and their genesis and abundance are regulated by coregulator complexes Integrator [100] and RNA Exosome [102], respectively. Employing NET-CAGE, which quantifies RNAs before they are affected by turnover, simultaneous generation of eRNA and mRNA was observed for the majority of the enhancer-promoter pairs [145]. In a novel cell-free assay that demonstrated enhancer-dependent promoter-driven transcription, we observed concurrent generation of both the eRNA and mRNA in vitro for the human *GREB1* locus [128]. The magnitude of eRNAs usually reflects the enhancer activity, measured as target mRNA levels [128, 137, 138, 144, 146, 147]. While it is unclear if eRNAs universally remain tethered to the enhancer chromatin or are released into the nucleoplasm [131], a polyadenylated subset of eRNAs reportedly can contact promoters in *trans* on a different chromosome and regulate transcription [148]. Additionally, nascent promoter-driven transcripts associate with enhancers [149].

To recap, not only an active enhancer itself undergoes transcription, but also the magnitude of its eRNA production mirrors the target promoter activity. Expectedly, physical contact is detected between an enhancer and promoter pair that are either dormant (not transcribing) or “active” (where both enhancer and promoter are producing eRNAs and mRNAs, respectively), but not when one is dormant and the other is active [150]. These observations led to the notion of functional connection among eRNAs, EPC, and promoter-driven transcription. Rather expectedly, several laboratories have reported that knockdown or overexpression of eRNAs correlatively affect the target gene expression and/or EPC [151]. Interestingly, the transcriptional impact of siRNA knockdown appears very specific [139]. This specificity is used to identify enhancers: the CRISPR-cas9 targeting is employed to recruit transcriptional repressors (CRISPRi) or activators (CRISPRa) to suppress or stimulate transcription at potential CREs, which leads to target gene modulation [152]. These considerations point to causal or coordinated transcription at an “in contact” enhancer and promoter pair. Indeed, transcription at cognate enhancer-promoter pairs is a highly coordinated process genome-wide [145], as well as in vitro [128]. We found that the *GREB1* enhancer and promoter fragments individually do not transcribe well, but undergo robust activation when co-incubated in conditions that support maximal EPC in *trans.* Using this system, we observed that it is the ‘act of transcription’ at the enhancer and promoter—but not the enhancer- or promoter-derived transcripts per se—that is mutually stimulatory [128]. That is, transcription-conducive nucleoprotein architectures at the enhancer and promoter stimulate each other’s transcription.

### Enhancer discovery

Several of the key features of enhancers described above are employed to identify enhancers. These approaches largely fall into five groups: (i) genome-wide maps of DNA accessibility; enrichment of epigenomic marks; occupancy of select TFs and TF clusters, coactivators and RNAPII; (ii) global transcription potential as assessed by GRO-seq, or mapping genomic TSSs by CAGE; (iii) assessment of transcription activation potential of regulatory sequences using reporter genes; (iv) targeted perturbation of the transcriptional status of the CREs by their suppression or activation; and (v) genome-wide assessment of chromatin connectomes to identify ‘in contact’ enhancer-target pairs [23, 153, 154]. Once an enhancer is identified, the next challenge is to determine its target gene. The straight-forward way is to quantify the known target gene activation upon enhancer perturbation [152, 155]. However, large-scale enhancer-target discovery efforts are usually plagued with varying degrees of off-target effects and multilateral regulatory control (i.e., multiple enhancers may regulate a target gene, or an enhancer may target multiple genes). Therefore, the best approach forward to conclusively identify functional enhancer-gene pairs is to integrate enhancer signatures with chromatin connectomes and underlying transcription in a defined population of cells [156]. Since transcriptional activation of a given gene is not synchronous among cells in a population in an identical environment, or even among alleles within a cell [157], accurate identification of functional enhancer-gene pairs will require integrative quantification of enhancer features, underlying transcription, and chromatin connectomes for each allele.

**Fig. 2:**
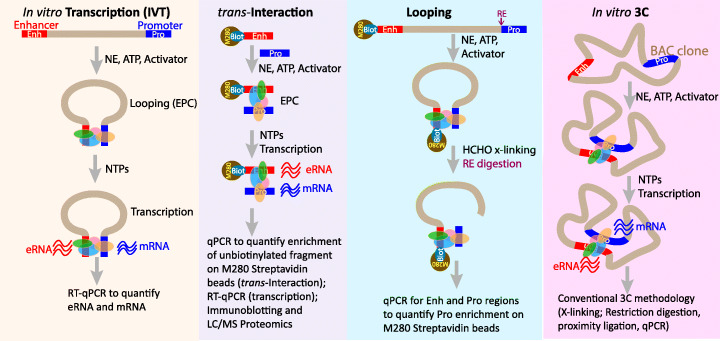
Cell-free assays to interrogate EPC and transcription. Four cell-free assays are envisaged where a construct with an enhancer and a promoter, or isolated enhancer and promoter fragments, or a BAC clone can be used as templates to interrogate EPC and transcription activation. Streptavidin-coated M280 magnetic beads are employed to capture EPCs on biotinylated fragments. These assays offer unique advantages not achievable through existing cell-based approaches, such as (1) same-source verification of EPC and transcription, (2) exploring mechanistic and/or causal link between EPC and transcription, (3) proteomics of EPC to identify protein complexes that (i) mediate looping and (ii) ensure transcription, and (4) biochemistry of EPC to elucidate requirements of non-proteinaceous ingredients (e.g., ATP, NTPs, coenzymes, and cofactors) in looping-coupled transcription activation. Arguably, any putative enhancer-promoter pair can be studied using the nuclear extract (NE) prepared from a relevant cell line. See (Panigrahi et al., 2018) for details

Cell-free methodologies [128] could offer an alternative approach for enhancer discovery and characterization. The cell-free assays involve two main components: (i) a construct with a natural enhancer-promoter pair with natural, shortened intervening sequence, or individual fragments containing the enhancer and promoter, and (ii) a protein source to support EPC and transcription, such as the classical Dignam nuclear extract [158] (see Fig. 2). We believe the EPC-coupled-transcription strategy to be versatile, which can be optimized for any enhancer-promoter pair activated by the required activator and using the nuclear extract from a relevant cell type. Since the biochemical compatibility between the proteomes at the enhancer and promoter is likely a critical factor determining enhancer-promoter interaction specificity [159], this approach could potentially uncover novel functional enhancer-promoter pairs.

## Mechanism of enhancer action

How exactly the enhancer activates target gene transcription is a persistent question in the field of eukaryotic gene regulation. To impart regulation, the enhancers must communicate with the target promoters. However, the enhancers in higher eukaryotes are physically separated along the genome from the target gene promoters. Several models have been proposed for enhancer-promoter communications. These include tracking (a transcriptional activator recruited to the enhancer tracks along the chromatin until it reaches the promoter), linking (an enhancer-bound transcriptional activator recruits proteins to for a chain that ultimately connects to the target promoter), and looping (proteins assembled at the enhancer and promoter make direct physical contacts) [21, 160, 161]. While the tracking model would be over-reliant on motor proteins and would impede the action of intronic enhancers (the tracking mechanics would collide with transcribing RNAPIIs), it is inconceivable that large chains of linking protein moieties would be employed genome-wide. As detailed below, the looping model enjoys overwhelming experimental support for establishing distal enhancer-promoter contacts (EPC).

### Detecting EPCs

As discussed above, enhancers are dense clusters of TFBSs that assemble large protein complexes representing various structural and functional classes, and the protein-enriched enhancers greatly activate transcription from specific target promoters. Arguably, such specificity can be achieved only through direct contacts between an enhancer and promoter pair, likely through protein-protein interactions [162, 163]. This notion initially was demonstrated by electron microscopy of a purified DNA template complexed with purified proteins that bound the enhancer or the promoter [164], modeled after studies of bacterial transcription mechanisms [165]. For the want of explorative studies within the cellular environment, a method was envisaged to detect two otherwise distant regions of DNA in close physical proximity. This methodology involved restriction digestion of DNA around bound proteins and ligation of the new DNA ends [166]. This methodology caused the advent of the chromosome conformation capture (3C) technique and its many derivatives [167, 168] and created the new discipline of ‘chromatin connectome’ that revolutionized our understanding of overall chromosome structural and functional dynamics [17, 169]. It is also now possible to achieve enhancer-promoter contacts (EPC) in vitro and simultaneously quantify both EPC and transcriptional readouts at the enhancer and promoter (Fig. 2), allowing direct functional studies into the mechanistic relationship among EPC, enhancer transcription, and promoter activation [128].

### EPC correlates with gene expression genome-wide

Employing the 3C-based connectome approaches or fluorescence-based visualization techniques, at individual loci or at genomic scale, studies over the past two decades have revealed several important features of enhancer action as a function of EPC (see Table 2). These studies provide strong evidence that EPC not only correlates with but also is required for transcription activation. Individual TFs and various protein complexes with disparate functionalities have been implicated in formation and sustenance of EPCs. Many enhancer-promoter pairs exist in preformed contacts in the absence of transcription, emphasizing that an EPC does not automatically guarantee transcription activation.

**Table 2:** Features of enhancer-promoter contacts (EPC)

EPC features	Select references
EPC is pervasive genome-wide	[170–174]
Two types of EPC	
Facultative (de novo): formed on demand for transcription	[128, 129, 144, 175, 176]
Preformed: keeps gene loci poised for transcription	[170, 177–180]
EPC correlates with active transcription	[128, 181–183]
EPC is required for transcription or “forced” EPC results in transcription	[184–187]
EPC is required for PIC recruitment, transcription elongation	[91, 188, 189]
Promoters in contact with active enhancers exhibit higher transcription rates	[170]
Sustained transcription is not required for the maintenance of EPC	[170, 190]
Proteins of various structural and functional categories can act as looping factors promoting EPC: tissue-specific TFs, Mediator complex, the Cohesin complex, CTCF; bridging factors LDB1, GATA3, OCA-B coactivator; chromatin remodelers SWI/SNF and NuRD; SRC-3; YY-1	[95, 128, 176, 187, 191–200]

### An EPC-transcription disconnect?

Despite both the profound conceptual logic and richness of data implicating EPC directly in transcription activation, there are reports that disagree [201, 202]. While it is possible that enhancers may adopt mechanisms other than direct EPC at certain loci, both technical and conceptual considerations can explain such observations. As discussed in the next section, EPC must be flexible and can be transient. Since transcription occurs in pulses, EPC need not be maintained after a burst of transcription initiation has occurred. Therefore, the best chance to capture a functionally competent EPC is during transcription initiation, which these two studies overlooked. Moreover, this could be a result of transcription-induced mobility of the enhancer within the nuclear space [203], an observation yet to be extensively tested in the context of EPC and transcription dynamics. Also, the above studies measure the gene-enhancer distance in fluorescence images at micrometer to sub-micrometer scale, though the enhancer and the promoter can be at the most 70 nm apart even with the most liberal estimates, which is beyond the scope of spatial resolution in many fluorescence imaging studies (for a comparison, up to twenty transcribing RNAPII complexes can reside in a cluster 70 nm in diameter [204]). Even as “seeing is believing,” not seeing an enhancer-promoter juxtaposition does not mean that EPC did not occur.

### Activation of transcription

#### Two steps in transcriptional activation

The above discussion establishes near-universal correlation between EPC and transcription, while also emphasizing that mere EPC does not automatically ensure transcription activation. Thus, some critical changes must occur at the EPC to trigger transcription. Evidently, enhancers can impact the promoter in many mutually non-exclusive ways, including reconfiguration of chromatin structure and modifications, recruitment of the pre-initiation complex (PIC), delivering RNAPII, removing repressors, and facilitating pause-release (see [205] for an extensive review). However, we still do not have a clear understanding of the actual mechanics of transcriptional ‘activation’, which essentially means rapid, multiple rounds of productive transcription from the promoter. Conceptually, enhancer-mediated transcription activation can occur in two broad steps: recruitment [206] and synergism [207]. First, sequence-specific TFs are recruited to the enhancer and promoter cooperatively, collectively, or additively, as discussed earlier (see [89] for an extensive review). Since the activation domains of TFs are intrinsically disordered regions (IDRs) with a high potential to interact with other proteins [208, 209], they further recruit other TFs and coactivators relevant to the transcription reaction (Fig. 1). For example, the coactivators SRC-3, p300, Mediator, and the MegaTrans complex of multiple TFs and enzyme activities are recruited to estrogen-responsive enhancers via pre-bound ERα [81, 83]. Second, the TFs display functional synergy; that is, the transcriptional output with two TFs is greater than the sum of the transcriptional output with the individual TFs, indicative of “functional amplification” of TF action. Transcriptional synergy has been demonstrated extensively, for example, in progesterone signaling [210], in TFIID function [211], in motor neuron specification [212], or in synthetic transcriptional circuits [213], while the detailed mechanics of synergism remains unclear.

#### Activation must involve amplification of TF action

Since TFs do not carry catalytic properties, their functional amplification can arguably happen in two mutually non-exclusive ways. First, a TF can undergo conformational change upon contacting a coregulator such that this interaction is specific but transient, allowing the coregulator to contact proteins at the promoter, after which the initial TF-coregulator contact is severed. This frees the TF for another round of coregulator recruitment and transfer, in cycles of association and dissociation. This model was well illustrated in yeast: transcriptional activation occurs when components of the GTF-RNAPII machinery are covalently connected to enhancer-bound proteins [214, 215], but not when the activation domain is transferred to the promoter-bound RNAPII machinery [216]. It is important to note that these association-dissociation cycles as described here may not necessarily reflect successive cycles of transcriptional events (i.e., RNAPII recruitment-transcription initiation-promoter clearance-transcript elongation; RICE); rather it may describe multiple dynamic interactions within the EPC during one transcriptional event. Also, these steps can be numerically amplified when multiple TFs are involved, resulting in transcriptional synergy (Fig. 1). Coregulators bridging the enhancer and the promoter may also undergo similar conformational or compositional changes. For example, the tail module of the Mediator complex interacts with the enhancer-bound TFs while the head and the middle modules contact the RNAPII and PIC at the promoter. Phosphorylation of the Mediator by TFIIH—which also phosphorylates RNAPII CTD to instigate transcription initiation—renders the Mediator-PIC interaction transient [98, 192]. These dynamic and transient links between the enhancer and the promoter—mediated by the Mediator complex—may *somehow* amplify the TF function (Fig. 1).

The second way to amplify TF action is through recruitment of catalytic activities. Since transcription itself is an enzymatic reaction, its activation must have enzymatic explanations. Thus, the enhancer-bound TFs can recruit enzymes that impact transcription reactions much the same way as coactivator recruitment, but with greater transcription output. The enzymes here might include kinases and phosphatases, acetyltransferases and deacetylases, methyltransferases and demethylases, ATP-dependent chromatin remodeling factors representing ATPases and helicases/translocases, etc. For example, TFIIS and p300 synergize to activate transcription [217]. These considerations establish that the TFs do not “act” in transcriptional activation per se; but they provide the requisite platform on which coactivators and other enzymes execute catalytic activities, leading to transcriptional activation.

#### Importance of EPC dynamism

The above discussion points to a critical feature of EPC function: that the contact between a given enhancer-promoter pair must be dynamic, and both formation and severance of contacts between an enhancer and its cognate promoter are important for eventual transcriptional activation (Fig. 1 [128]). In a subset of ERα-dependent genes, failure to recruit the steroid receptor coactivators impairs estrogen-induced transcription activation despite a substantial increase in EPC [81], as mirrored in a scenario of SRC-3 depletion [128]. This activation defect may be partly because of a rigid EPC where the enhancer and promoter fail to dynamically dissociate and re-associate, abrogating the possibility of transcriptional synergy. This notion of dynamic EPC in transcription activation is supported by the observations of transcriptional bursts. By transcriptional activation, we essentially mean higher production of the desired transcripts per unit time, when the available template molecules are a constant. In a population context, such higher production of the given transcript can occur if more templates undergo steady transcription, or a few templates undergo rapid and multiple rounds of transcription in “bursts.” It is now recognized that most genes in various model systems transcribe in bursts, followed by prolonged periods of inactivity [218]. Bursts can happen by increasing the number of transcribing RNAPII per burst (referred to as burst size or amplitude), which is governed by the promoter architecture [219]. Here, key promoter-proximal TFs dwell longer at stronger promoters, allowing more transcriptional events per burst [220]. Alternatively, burst frequency can be increased without impacting the amplitude. Increased frequency of bursts is largely a function of the enhancer strength [219, 221]. It is known that enhancers increase the probability of transcription, and not the level of transcription [222]. Thus, a strong enhancer likely contacts the promoter more frequently, each contact perhaps representing one transcription event. Such a probabilistic scenario is possible only if EPC is not a stable, enduring connection, but instead is a dynamism of contact formation and severance [128, 184]. The preformed EPCs detected genome-wide at transcriptionally inactive gene loci represent a state of poised enhancer-promoter proximity that is still waiting for the defining reaction to occur to kick start transcription [223], and hence the dynamism of EPC formation and severance.

### Controlling enhancer action through chromatin organization

It would be nearly impossible for an enhancer to find a cognate promoter if the entire genome is randomly and homogenously dispersed in the nuclear space, with no substructure. However, the chromosomes as large soluble polymers impede free intermingling of themselves in the nucleus. This forces them to assume distinct chromosome territories such that there is little contact between chromosomes [224, 225]. Each chromosome is further structurally organized into several compartments of the A type (largely transcriptionally active) or B type (largely transcriptionally silent). Regions in compartment B exhibit higher interactions within, indicative of a more compact structure, than those in compartment A [225]. Each compartment is further organized into topologically associated domains (TADs) where there are minimal interdomain interactions and maximal intradomain interactions; TADs in vertebrates are demarcated by CTCF-bound insulator elements [226, 227], where Cohesin generates the TAD architecture by loop extrusion [228–230]. Clusters of non-coding CREs and target genes, along with bystander genes have been preserved in evolution to spatially coexist as genomic regulatory blocks (GRBs) [34]. The GRBs have been identified as TADs in as diverse organisms as *Drosophila* and human [231], indicating deep evolutionary roots of TADs. An enhancer and its cognate target gene almost always reside within a TAD; i.e., an enhancer’s functional jurisdiction is largely limited to the home TAD as it seldom contacts genes residing in neighboring TADs [232, 233]. Sequence alterations at the TAD boundaries can restructure the adjacent TADs such that an enhancer can contact and activate a gene that resides in an otherwise inaccessible TAD, best illustrated in developmental disorders [234, 235]. This spatial restriction eases the effort for the enhancer and cognate genes to find each other. However, not all promoters and enhancers within a TAD interact with each other, and there exists precise enhancer-promoter selectivity (see below).

Cohesin appears to play a very critical role here: the STAG1-containing Cohesin creates and maintains TADs as anchors of “macro” inter-TAD loops, whereas the STAG2-containing Cohesin mediates the ‘micro’ intra-TAD loops necessary for establishing EPC [236]. It is important to note that even as Cohesin depletion appears to abolish the TAD structures at population scale, the impact on over-all transcription is minimal [237]. Single cell experiments reveal that specific TADs survive Cohesin loss [238]. Combinedly, these observations suggest that Cohesin loss restructures the TADs such that the distinctiveness of the TADs is not detected at a population scale. Therefore, it is possible that additional mechanisms other than Cohesin-mediated loop extrusion contribute to chromosomal architecture and enhancer function [239].

### Complexity of multi-enhancer control

It is now established that the number of CREs in the metazoan genome outnumbers the genes [138, 240]. Research into capturing promoter-centric or enhancer-centric interactions have revealed not only the usual suspects, i.e., enhancer-promoter interactions, but also numerous enhancer-enhancer, promoter-promoter, enhancer-gene body, and promoter-gene body contacts [240, 241], despite distinct network architectures of the enhancers and promoters [173]. Although it is unclear what fraction of these interactions are functionally relevant in any given cell type, what is beyond dispute is that an average gene is under the control of multiple enhancers [242]. For many genes, it has been demonstrated that redundant enhancers allow temporally specific as well as quantitatively precise gene expression and may compensate for the loss of other enhancers, thereby conferring phenotypic robustness [243]. The multiple enhancers for a given target gene may be classified as predominant or supportive, where the latter becomes functional upon inactivation of a predominant enhancer [244]. According to a new report, the most distal enhancer in an enhancer chain usually acts as the predominant regulator of a target gene [245].

So, in such multi-enhancer systems, what determines which enhancer to contact the promoter and when? This problem is also relevant to the EPC selectivity within TADs discussed above. For every enhancers and promoters, the underlying TFBSs dictate TF occupancy and eventual recruitment of coregulators. The biochemical compatibility imparted by the different protein complexes assembled at different enhancers and promoters can potentially explain enhancer-promoter interaction specificity [246–249]. Recently, transcriptional specificity of several *Drosophila* coregulators bound to a proximal enhancer has been worked out for different core promoters, revealing different compatibilities [250]. An extension of this technology can potentially reveal TF-coregulator compatibilities for distal enhancers and distinct promoter types.

## Emerging concepts in enhancer action

### Enhancer or silencer?

Since the chromatin structure itself is an overwhelming impediment to transcription, research on transcriptional activation mechanisms has been tremendous. However, there are position- and orientation-independent silencer elements in the eukaryotic genomes that mediate active repression of transcription [251, 252]. With the premise that active silencers (i) would be nuclease-sensitive due to assembly of repressor protein complexes—as are the enhancers, (ii) would be enriched with repressive epigenomic marks such as H3K27me3, and (iii) would suppress transcription of the neighboring genes, thousands of silencer elements have been recently discovered in multiple human cell lines [253]. These silencers are enriched with binding motifs for many repressors; several of these silencers were also experimentally validated in reporter assays. Another study focused on previously uncharacterized nuclease-hypersensitive CREs enriched with repressive TFBSs and employed massively parallel reporter assays that identified thousands of silencers in multiple human and murine cell lines [254]. Several of these elements were found in contact with inactive genes in published Hi-C datasets.

Just as an enhancer is not supposed to be active in all cells, so is a silencer expected to be active in specific cell types. Also, just as an enhancer can be inactivated through the recruitment of repressive proteins [255, 256], a silencer can also be potentially deactivated. Thus, depending on the presence of coactivators or corepressors, the functional identity of the enhancers and silences may potentially switch. Interestingly, several silencers reportedly act as enhancers in different cell types [254, 257]. We envisage six scenarios emerging from these studies: direct activation and repression by enhancers and silencers, respectively; passive suppression and expression upon dismissal of coactivators and corepressors from enhancers and silencers, respectively; and enforced reversal of enhancer and silencer functions when they gain repressors and activators, respectively. Whether such scenarios indeed play out await experimental evidence.

### Condensates encompassing EPC

**Fig. 3:**
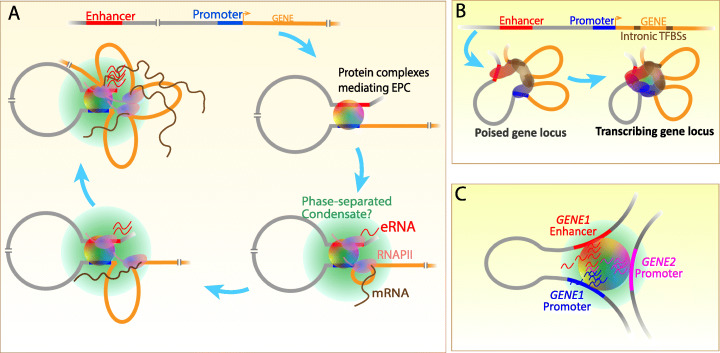
A schematic for transcriptional coordination between the enhancer, promoter, and gene body. **a** Multiple protein complexes specializing in many structural and catalytic activities establish EPC. RNAPII recruitment and transcription initiation occur in a phase-separated condensate (a “transcription bubble”) where productive transcript elongation takes place. RNAPII remains within the EPC-encompassing transcription bubble, necessitating extrusion of the downstream template DNA into a loop behind—as elongation proceeds. Sequential recruitment of RNAPII represent transcription of the gene in multiple transcription units, each forming a DNA loop as in the petals of a sunflower. **b** Additionally, the chromatin landscape of a transcriptionally poised gene can exist in a sunflower arrangement where proteins assembled at intronic TFBS clusters hold the enhancer and promoter in proximity without direct EPC. Transcription initiation accompanies direct EPC. This model can supplement and co-operate with (**a**). **c** Multiple gene promoters can exist in physical proximity with a given enhancer within a phase-separated condensate, facilitating coordinated transcription activation. Likewise, a given promoter can also exist in association with multiple enhancers simultaneously

Over the recent years, it has become increasingly clear that various biochemical reactions undergo liquid-liquid phase separation (LLPS) into condensates inside the cell, where not only the efficiency and fidelity of biomolecular processes are enhanced, but also the organizational and architectural specificity is attained without requiring membranous confines [258, 259]. Two factors are important for phase separation of molecules: a network of interactions among the participating molecules so that the local concentration is effectively high; and the surface of the molecules conducive enough to support such interactions—for example, a multi-modular feature called “multivalency” [260], where each module can potentially initiate interactions. Examples of multivalent interaction surfaces in macromolecules include unstructured patches or IDRs in proteins, the modular structure of RNA, high TFBSs as in enhancers, etc. Multivalency can spontaneously initiate oligomerization that soon results in a polymer, which forms high-density liquid droplets upon LLPS. A critical feature of these liquid droplets is the exchange of molecules with the surroundings. This provides an ideal platform for multicomponent biochemical reactions to take place. Transcription is one such molecular process, and LLPS was theorized to regulate transcription [261]. Subsequent studies have now demonstrated that the Mediator complex, various TFs with IDRs, coactivators, and RNA Polymerase II (RNAPII) form condensates during transcription [208, 262–265]. Multivalent DNA sequences on enhancers also promote condensations of the bound TFs and coactivators through LLPS even at moderate concentrations [266]. LLPS is reported to cause enhancers to come closer that otherwise reside in faraway TADs [267], although this notion has not been tested rigorously. Nevertheless, these studies have established that phase separated condensates form on enhancers and correlate with transcription activation. Taking into account the various components of transcription regulation undergoing LLPS, it is safe to assume that the RNAPII-Coactivator condensates encompass EPCs (see below; Fig. 3), though definitive proof is desired. A recent study suggests that local RNA concentration can regulate condensate formation and dissolution, thereby functioning as a transcriptional feedback mechanism [268].

### Transcription bubble at EPC

The relative motion of the template DNA vis-à-vis the elongating RNA polymerase is an area of active debate. There are two possibilities: the RNAPII leaves the promoter after transcription initiation, pauses, and “tracks forward” along the template DNA like a locomotive as the RNA synthesis progresses. Alternatively, the RNAPII can stay tethered to nuclear structures while the template DNA “extrudes backward” during chain elongation. Nearly 40 years ago, Peter Cook and colleagues observed that the “body” of nascent RNAs are tethered to nuclear structures—presumably through the RNAPII itself [269]. This led to the “extrusion backward” model, while the “tracking forward” model enjoys general acceptance. From a cellular perspective of population energetics, it would be wasteful to engage numerous RNAPIIs along numerous genes discretely such that at any given time thousands of transcribing genes populate the entire nuclear space. A more productive way would be to have transcription factories [204], where congregations of genes could be transcribed in a concerted fashion. In fact, transcription-associated RNAPII condensates provide support to this view [262].

Over the years, many genome-wide [170, 241, 270] and locus-specific [271, 272] studies have observed promoter-gene body contacts that also associate with RNAPII. This is possible only if the RNAPII remains in contact with the promoter and downstream transcribed regions simultaneously. In that case, the only way for the RNAPII to elongate the RNA chain is to drag the template backward, causing DNA extrusion. Indeed, Blobel and colleagues observed the enhancer, the promoter, and the progressive regions of the gene body held together in close proximity as transcription continued [273]. While studying the transcription kinetics of the *GREB1* locus, we observed that two gene body SRC-3-bound regions (GBS1 and GBS2) hold the enhancer and promoter in close proximity in estradiol (E2)-deprived MCF-7 cells. However, upon E2 stimulation, the GBS1 and GBS2 (18 kb and 43 kb downstream of the promoter, respectively) promptly disengage from the enhancer allowing direct EPC, and they then get back in contact with the EPC when their respective regions are undergoing transcription [128], supporting the extrusion model. We made similar observation at the *NRIP1* locus as well. Recently, a strong proof for this model has come in the form of “stripes” in an exemplary nucleosome-resolution “Micro-C” connectome of the transcription-linked chromatin [274]. The stripes extend from the promoter and cover the entire length of active genes, which is possible only if the samples represent the promoter’s contact with the body of the gene progressively.

Taking the above observations along with the recent transcriptional LLPS studies, we propose that the enhancer and the promoter reside within a phase-separated “transcription bubble” enriched with coactivators and the RNAPII, and that transcription elongation occurs when the downstream template DNA is dragged backward causing a progressively extruding loop (Fig. 3a). A recent global RNA-RNA interaction map has revealed physical proximity between eRNAs and transcripts derived from target promoters [275]. The experimental methodology in this study rules out detection of RNA-RNA interactions in free-floating ribonucleoprotein particles, emphasizing that the specific eRNA-mRNA contacts are chromatin-mediated and, thus, recapitulate EPC. Such direct contacts between the chromatin-anchored transcripts are possible only when the enhancer and the promoter reside in a transcription bubble during transcription (Fig. 3). It is also possible that strategic gene body locations within the gene, like the SRC-3 enriched GBSs, may exist as preassembled TF-Coactivator hubs. In fact, more than half of all genomic TFBS clusters reside in intronic regions [276] and many genes contain SRC-3 enriched GBSs that coincide with the intronic TFBS clusters [277]; however, their functional relevance has not been explored. We envisage that these intronic TFBS clusters assemble into bound TF-coactivator hubs to aid the enhancer-coordinated transcription of the gene in a dynamic architecture akin to the petals of a sunflower (Fig. 3b). In our opinion, such a scenario can explain the concordance of transcription seen at the enhancers and target genes, coordination of enhancer-regulated transcription of the gene body, observation of multi-enhancer and multi-promoter contacts as observed at genomic scales [170, 241], as well as simultaneous regulation of more than one gene by a single enhancer (Fig. 3c) [221].

### Intronic regulation of enhancer action

An offshoot of the transcription bubble and the sunflower model described above is the tantalizing prospect of intronic regulation of enhancer action. Since the enhancer, promoter and the strategic intronic TFBS clusters (e.g., GBSs) maintain near-constant contact, these nucleoprotein hubs can structurally and functionally influence each other (Fig. 3b). Interestingly, not only the genomic interactions of GBS1 and GBS2 with the *GREB1* enhancer and promoter are recapitulated in vitro, inclusion of GBS1 and GBS2 fragments greatly enhance cell-free transcription of both the enhancer and promoter [128]. These results would suggest that GBS1 and GBS2 might be acting as intronic enhancers, but these elements do not exhibit any recognizable epigenomic signatures of active enhancers [144]. Thus, it is likely that strategic intronic TFBS clusters constitute a novel functional class of gene regulatory elements that not only regulate the EPC, but also impact transcriptional processivity.

## Unanswered questions on enhancer action

### Why, how, when to contact a promoter

Despite the extensive information about enhancers as sampled above, our mechanistic understanding of enhancer function remains incomplete. The key question of *how exactly enhancers activate promoters* still awaits definitive answers. The mechanism of enhancer action as discussed above—that enhancer-bound TFs help recruit other TFs, GTF, coactivators, and the RNAPII holoenzyme at the promoter to stimulate transcription—is predominantly based on our knowledge of the transcription regulation in yeast, *Drosophila,* and mammalian model systems where the enhancer elements are in close proximity to the promoters [278]. While the basic biochemical steps in transcription remain the same in yeast and higher metazoans, the latter have evolved significant differences, including more complex regulatory sequences, larger repertoire and diversity of TFs and coregulators, additional transcription-associated epigenetic signatures, greater enhancer-promoter genomic distance, and greater hierarchy of chromosomal organization [279]. We do not as yet have a systematic, detailed, and integrative molecular understanding of how these features impact mammalian transcription.

As discussed earlier, EPC is required to transmit the regulatory instruction from the enhancer onto the promoter, and yet an EPC does not guarantee transcription. Arguably, it is useful to have a preformed EPC(s) so that the gene locus is poised for transcription—waiting for a final kick start. But, what determines which set of enhancers and promoters should stay in preformed contacts, when, and for how long? Are there any universal norms that govern genome-wide EPC at non-transcribing loci, or do individual EPCs have their own molecular explanations? Are there any definitive and universal looping determinants that mediate EPC at all loci (even the Cohesin and the Mediator complex can be dispensable for EPC [280, 281])? Or, is EPC a result of concerted efforts of many TFs and coactivators, whose detailed identities may be locus-specific? Further, why do the enhancer and promoter move apart during transcription in a few cases [201, 202]? Are these observations a result of conceptual and procedural inconsistencies, or is there an alternate pathway to transmit the enhancer-encoded instructions onto the promoter, without involving any physical contact? An idea of indirect contribution of RNAPII and Mediator in EPC—by somehow making the looping chromatin interfaces accessible to architectural proteins—has been floated [281], but has not been adequately tested. Also, most studies employing fluorescence in situ hybridization (FISH) approaches with ~ 50 kb-long probes show the enhancer and promoter at distances greater than hundreds of nanometers, yet consider them as “in physical proximity.” These distances are nearly an order of magnitude larger than an estimated distance between any given “in contact” enhancer-promoter pair along with the assembled proteomes. Then, what actually do we mean by physical proximity? Can the physical contact between the proteomes assembled at the enhancer and promoter be still possible at such great distances? Or, can a huge phase-separated transcription hub, encompassing a congregation of multiple gene loci, explain such supposedly direct-yet-distant regulation?

### After contacting a promoter

Since mere EPC does not automatically ensure transcription, the preformed EPCs raise another critical question: what happens after an enhancer contacts a promoter? What triggers the transcription? Detailed genetic, biochemical, genomic, structural, and proteomic investigations into the stepwise transitions in transcription, from TBP binding and PIC assembly to promoter clearance, pausing, pause release and productive elongation, have generated a tremendous volume of information [278, 282–284]; however, it is unknown which critical reactions and players act as the defining trigger for the EPC in first becoming transcriptionally competent, and then succeeding in productive chain elongation. It was suggested that EPC can help recruit RNAPII and associated factors to the promoter to facilitate transcription initiation [91, 163] and elongation [188, 273], which apparently are not enough to trigger transcription. Not only that, RNAPII occupancy is already detected at EPCs in the absence of transcription stimulation [144], indicating that the presence of RNAPII cannot be the defining trigger either. Since the predominant mode of transcription is bursting and enhancers primarily increase the burst frequency [184, 219, 285], transcription “activation” is likely accomplished by an increased pace of RNAPII “recruitment-initiation-clearance-elongation” events (RICE), which conceivably involve both compositional and structural reorganization of the EPC during successive rounds of transcription. This raises the question: what are the molecular determinants and definitions of such reorganization?

Answering these questions would require the development of new methodologies. Arguably, isolation of genomic EPCs at sufficient temporal resolution during the transcriptional transitions and comprehensive proteome analysis thereof could provide deep insight into these questions. However, this is a formidable task because, first, we currently do not have an easy way to isolate locus-specific EPCs from the genome in sufficient quantities that would allow such detailed investigations [286]; preparing a single sample for a given genomic EPC would require more than a billion cells. Second, the cellular environment presents some challenges that impede deeper mechanistic studies. For example, it is impossible to address the proteome-based mechanism of burst dynamics as discussed above, since the bursts are cell-intrinsic and are the source of ultimate transcriptional heterogeneity in a population. Third, addressing many of the questions would require controlling the availability of not just the stimulating signals and the key transcription factors – which is relatively easy to accomplish, but also of ATP, which is not possible in a cell-based model system. Fourth, the current interrogation of EPC and transcription through approaches based on 3C/DNA-seq, RNA-seq, and fluorescence (FISH) each adopt very different experimental pipelines, and the resultant interpretations of the various types of data are correlative at the best. Therefore, the best option towards deeper mechanistic studies on EPC-transcription dynamics may be to employ cell-free methodologies for EPCs coupled with transcriptional readouts and proteomics (Fig. 2).

### Why eRNAs?

The fact that eRNAs are produced genome-wide in animals as diverse as *C. elegans*, fruit fly and mammals raises the possibility that enhancer transcription is a product of transcriptional noise. However, the same observation also argues that it might be a conserved phenomenon with a purpose. This latter possibility is supported by the fact that full complements of GTFs, RNAPII, and other critical coactivators occupy active enhancers, suggesting that the resultant eRNAs might have a cellular function. The considerations described in the earlier section arguing mutual coordination between the enhancer and promoter transcription further raise the questions: *why are enhancer and promoter transcription coordinated*, and what is the molecular basis of that coordination? As discussed above, the “act of transcription” at an in-contact pair of enhancer and promoter is mutually stimulatory. What is the biochemical explanation of such mutualism? The subsequent questions that emerge are: why must an enhancer transcribe, and do eRNAs have any generalizable functional relevance?

The eRNAs have clearly emerged as an indisputable marker of active enhancers, and many in the field consider eRNAs as either a spontaneous byproduct of enhancer activation, or a ploy to keep the enhancer chromatin open (reviewed by [19, 140, 151]). Likewise, eRNAs have been reported to exhibit specific functional roles [141, 151, 287], which include helping establish an open chromatin structure to support transcription [288], recruiting Cohesin [148, 175] and Mediator [289], entrapping the negative elongation factor NELF [290] or YY1 [291], stimulation of histone acetyltransferase activity of the coactivator CBP [292]. Contrary to near-universal correlation of eRNAs and transcription activation, a recent report implicates eRNAs in nucleating ERα-dependent transcription repression [293]. However, the eRNAs across a genome differ both in sequence and structure, and hence must differ in functional specificity, if any. Therefore, none of these suggested functions can be generalized for all eRNAs, indicating that the universal function of eRNAs, if any, is still elusive. In addition, the proposed functions of eRNAs do not explain a more critical and pertinent question: why and how does the enhancer start transcribing eRNAs? We do not seem to have definitive answers to these questions yet.

## Conclusion and future direction

Enhancers carry the regulatory instructions for a gene’s spatiotemporal expression. The enhancer-encoded instructions are communicated to the cognate gene promoters via dynamic protein-protein interactions that involve multitudes of TFs, coregulators, chromatin architectural proteins, and enzymes. Moreover, many of these proteins acquire or shed covalent modifications necessary for proper transcriptional regulation. In concert with the promoter-driven transcription, the enhancers themselves also undergo transcription, producing eRNAs of uncertain functions. These multifaceted interactions among the underlying DNA sequences at the enhancer and promoter, the assemblies of proteins, and RNA molecules likely occur in phase-separated condensates. However, global or locus-specific dynamics of these multifarious interactions leading to productive transcription are still poorly understood. The current dogma posits that the enhancer-encoded instructions are translated into a ‘TF code’ when a certain combination of TFs binds the enhancers both directly and indirectly. This combinatorial TF recruitment to the enhancers is currently thought to define the transcriptional regulation of a target gene. We opine that the combinatorial TF occupancy merely provides a platform for dynamic recruitment of the coregulators, which ultimately govern transcriptional specificity and processivity. For a more holistic and mechanistic understanding of enhancer function, it is now the time to transition from the current “TF code” of transcription to a ‘coregulator code’, where the emphasis would be to define at high temporal resolution the combinatorial coregulator repertoire that drive locus-specific transcription activation.

## Supplementary Information

**Additional file 1.** Review history.
