# Supplementary material for: Mechanisms of enhancer action: the known and the unknown
Source: Genome Biol. 2021 Apr 15;22:108. doi: 10.1186/s13059-021-02322-1 (PMC8051032; doi:10.1186/s13059-021-02322-1)
Supplement: Supplementary file 1 — Additional file 1. Review history. [file 13059_2021_2322_MOESM1_ESM.docx]

Review History

**First round of review**

**Reviewer 1**

**Comments to author:**

I read with great interest the review manuscript by Panigrahi et al. entitled "Transcriptional Enhancers: the known and the unknown". In this manuscript the authors cover quite extensively what we currently know as well as some major open questions regarding the mechanism of action of enhancers. Overall, the manuscript is highly relevant and timely, as during recent years there have been major findings regarding enhancers that need to be discussed and put into the context of previous literature and proposed models. I definitely support publication, although I have some comments and suggestions that I hope the authors find useful.

-	In my opinion, the authors are a bit too strong and dogmatic about the necessity of enhancer-promoter contacts and physical proximity for enhancer function. The authors should acknowledge other mechanisms of action that have been previously proposed (tracking, linking, etc). In this regard, do the authors think that proximal (within a few Kb of a promoter) and distal enhancers control gene expression through similar mechanisms?. For proximal regulatory sequences we don't have many evidences of looping because current 3C-based or imaging methods do not have the resolution to detect such short-range interactions. Perhaps tracking or linking models can explain how proximal enhancers work. Moreover, recent work from Levine and others, mostly in Drosophila, suggests that distal enhancers might control gene expression through a combination of looping and tracking/linking mechanisms: long-range loops can bring enhancers close to a promoter, but not in direct proximity, and tracking or linking could bridge the distance.

-	The authors briefly mention at the beginning of the manuscript the main types of cis-regulatory elements, including enhancers and silencers. Later in the text, they state the following: "Proteins assembled at enhancers can promote either activation or repression of target genes depending on their repertoire of coactivator or repressor complexes, respectively." In my opinion, this statement can be quite confusing, since an enhancer that represses its target genes due to the action of co-repressors could define as a silencer. In fact, previous as well as recent work in Drosophila that same regulatory elements could work as either enhancers or silencers depending on the proteins bound to them (Gisselbrecht et al., Mol Cell, 2020). Moreover, during the last couple of years there have been a number of papers describing genome-wide identification of silencers, although their mechanisms of action (including whether or not they loop to target promoters) remain quite obscure. I think the authors should have a dedicated section to comment about the differences between enhancers and silencers, maybe in the "Emerging concepts" section.

-	Regarding the "Emerging concepts" section, another topic that I missed in the review was the mechanisms implicated in the control of enhancer-gene compatibility. Accumulating evidences suggest that not all enhancers and genes located within the same TAD or regulatory domain (see previous work and recent preprints from Lenhard, Furlong and Rada-Iglesias laboratories) can physically and functionally communicate with each other. Moreover, there are also emerging evidences of how the genetic composition of both promoters and enhancers as well as the protein complexes recruited to them can control gene-enhancer compatibility (see recent work from Alex Stark lab as well as Pachano et al. bioRxiv, 2020). On the other hand, and as mentioned above, dogmatic statements such as "An enhancer and its cognate target gene always reside within a TAD", since there are some evidences of enhancers acting in trans, including transvection mechanisms in flies. Moreover, TADs might not be a universal of metazoans genomes, while enhancers, as stated by the authors are likely to be so.

-	The authors dedicate quite some effort to describe the importance of enhancer-promoter contacts and how this can contribute to enhancer function and transcription. However, I think the authors should better describe the proteins and protein complexes that have been describe so far as regulators of enhancer-gene interactions both in the active as well as poised states.

-	Regarding eRNAs and the strong coupling between enhancer and gene transcription, I think the authors should discuss previously proposed models suggesting that both enhancers and gene promoters can act as independent transcriptional units (see work from Andersson and Sandelin lab). Moreover, the authors should also mention previous work suggesting that enhancer and gene transcription are mutually dependent i.e. eRNAs are not produced if gene promoters are eliminated or not compatible (Kim et al, Nature, 2010; Pachano et al, bioRxiv, 2020).

Minors comments:

-	When mentioning super-enhancers the authors should acknowledge conceptually similar concepts that were previously described and that have been extensively investigated, such as Locus Control Regions (LCRs).

-	Enhancer redundancy might contribute not only to transcriptional robustness but also transcriptional precision.

-	"The TFBSs in the majority of enhancers are modular in nature". Do we really have evidences to claim that the majority of TFBS are modular?. I don't think that enough enhancers have been genetically dissected with sufficient spatial resolution to make such a claim. Several models (enhanceosome, billboard, TF collective) should perhaps be briefly presented.

-	"That is, transcription-conducive nucleoprotein architectures at the enhancer and promoter stimulate each other's transcription. The question, then, is what comes first". Is there a need for an order? Could transcription at enhancers and promoters happen simultaneously?. This could be the case if they are mutually dependent rather than if they stimulate each other.

**Reviewer 2**

**Comments to author:**

The manuscript by Panigrahi and O'Malley presents a review of literature and ideas about enhancers and enhancer-promoter interactions. It is less about enhancers per se and more about what is known about enhancer-promoter complexes (EPCs), which is not a bad thing. I have enjoyed reading it and learned a lot. It is refreshing to read a review written not by one of the "usual suspects" in regulatory genomics, but by researchers developing novel in vitro systems for mechanistic studies of enhancer-promoter interactions. The authors know both the regulatory genomics and mechanistic literature very well. Their choice to put the emphasis on their own angle provides us from the genomics side with a fresh perspective and pointing to many important studies that I suspect many of us will have missed. At the same time, they integrate the discussion of those studies with the results from genomics that we are familiar with.

Given the angle of the manuscript, the title is a bit too broad - "Enhancer action" or "Mechanisms of enhancer action" would be a better match than "Transcriptional enhancers". In the latter case, most Genome Biology readers would expect to find a more conventional treatment of genomic features of enhancers.

Specific issues, both major and minor:

- p.3, line 57 "Caspaspora" -> Capsaspora

-p.3, "Moreover, microsynteny is evidently conserved across metazoans (ref. 31), indicative of the presence of enhancers in the bystander genes." - the concept of bystander genes vs target genes is part of the Genomic Regulatory Block (GRB) model, introduced in Kikuta H et al. 2007. Genomic regulatory blocks encompass multiple neighboring genes and maintain conserved synteny in vertebrates. 17: 545-555. I think that the GRB model deserves a proper mention in a review about enhancer-promoter interactions, especially since GRBs has since been shown to coincide with the strongest and most deeply conserved TADs devoted to regulation of developmental genes (Harmston N al. 2017. Topologically associating domains are ancient features that coincide with Metazoan clusters of extreme noncoding conservation. Nature communications 8: 441.). The GRBs and corresponding TADs are spanned by clusters of dozens of enhancers, many of which are ultraconserved, and include both the target genes of those enhancers as well as non-responsive bystander genes.

- p.4, line 18 "The TEs can navigate genomic loci far and wide" - I don't understand what this is supposed to mean.

- p.4, line 26 "Genomic duplication events, fractionally or in whole" - Strange wording. Try e.g. "Genomic duplication events, be it tandem duplications of chromosomal regions, duplications of whole chromosomes, or whole genomes". Also, in most cases it is moot which of the two copies is "new".

- p.4, line 53-54 "protein-coding sequences have diverged less compared to the non-coding sequences" - across certain evolutionary distances, this is not entirely true. Within e.g. vertebrate clade, ultraconserved sentences are non-coding sequences that often overlap developmental enhancers and are much more conserved than even the best conserved protein-coding sequences (there are almost 500 such sequences that are identical over 200bp or more between human and mouse, and there are hundreds that are extremely highly conserved between mammals and fish).

- p.5, "enhancers such as the human IFNbeta1" - IFNbeta1 is a gene, not an enhancer, you mean an enhancer that regulates IFNbeta1

- p.6, line 4: While "evolutive" is a legitimate word, it is not normally used in biology. Use "evolutionary constraints".

- p. 6, line 34-36, and Table 1: Why are reader proteins mentioned as part of the proteome of the enhancers, and writers and erasers are not? E.g. p300 is a writer and is definitely found bound to active enhancers.

- p. 7, line "the RNAPII and GTFs comprise a sum of 45 proteins" - the exact number and composition depend on the promoter class, cell type, and species.

- p.7, line 28: "inevitable role" - you probably mean "indispensable".

- p.8, line 14: the idea of "housekeeping enhancers" is still controversial - both in the case of ETS-bound elements (Curina et al.) in mammals and the STARR-seq detected "housekeeping enhancers" in Drosophila (Stark lab), the elements are so close to core promoter that it is equally possible to consider them part of the promoter architecture, and not separate enhancer elements.

-p.8, line 54-56: "Widespread intergenic transcription was detected at genomic scale twenty five years ago and such pervasive transcription was soon mapped to enhancers" - this sounds like enhancer transcription is the main/dominant cause of intergenic transcription. On the contrary, much of the intergenic transcription was due to previously undetected noncoding RNAs (lncRNA and some shorter classes) whose transcription initiation is not thought to universally start at enhancers.

- p.8, line 60 - p.9, line 4: "Studies employing CAGE have estimated that over 43,000 distinct eRNAs can be detected in a human cell" - this is not quite right. First, the study in question (Andersson et al. 2014) defines enhancers as having near-symmetrical bidirectional CAGE signal, so 43,000 enhancers would equal twice as many (86,000) eRNAs. Second, they were not detected in a human cell, but in a cumulative signal from almost 1000 CAGE samples. Third, the number is depth-dependent, and subsequent CAGE experiments detected even more enhancers with bidirectional eRNA signatures.

- Regarding the above, it would be important to mention an alternative explanation of eRNA CAGE signal: see Young RS, Kumar Y, Bickmore WA, Taylor MS. 2017. Bidirectional transcription initiation marks accessible chromatin and is not specific to enhancers. Genome Biol 18: 242.

- p.9, line 16-17: "lipopolysaccharide- induced eRNA synthesis in mouse macrophages precedes transcription from the target promoter" - while this is the conclusion of Arner et al Science paper, the subsequent NET-CAGE paper reveals it to be a likely artefact of the fact that regular CAGE detects mature RNA, and that eRNA is much less stable than the target promoter mRNA, resulting in faster accumulation of eRNA than mRNA to the steady state levels after simultaneous induction.

- p. 11, line 37: "since the regulatory control of gene expression in higher eukaryotes is essentially uncoupled from the act of target gene transcription per se, it is imperative that these two steps are connected" - I am not sure what this means; it reads like "since X and Y are uncoupled, it is essential that they get coupled". But if they get coupled then they are not uncoupled. Please rephrase.

- p.16, line 32-34: "TADs are demarcated by CTCF-bound insulator elements" - that is not universally true for all Metazoa (e.g. Drosophila).

- p. 17, line 4: "Cohesion-mediated" -> "cohesin-mediated"

- p. 17, line 30-32: "According to a new report, the most distal enhancer in an enhancer chain usually acts as the predominant regulator of a target gene (ref 236)" - Based on what ref. 236 actually shows and how it shows it, I would not make such a strong claim out of it.

- p.17, line 51: "a multi-modular structure called 'multivalency' " - please cite reference where this structure is first suggested (it is not clear at the moment).

- p. 18, line 41: "Such direct contacts between the chromatin-anchored transcripts is possible" -> "... ARE possible"

**Authors Response**

**Point-by-point responses to the reviewers’ comments:**

Reviewer #1: I read with great interest the review manuscript by Panigrahi et al. entitled "Transcriptional Enhancers: the known and the unknown". In this manuscript the authors cover quite extensively what we currently know as well as some major open questions regarding the mechanism of action of enhancers. Overall, the manuscript is highly relevant and timely, as during recent years there have been major findings regarding enhancers that need to be discussed and put into the context of previous literature and proposed models. I definitely support publication, although I have some comments and suggestions that I hope the authors find useful.

*We thank the Reviewer for appreciating our work. The Reviewer also has made the comments and suggestions that improved the manuscript. We have incorporated the suggestions.*

- In my opinion, the authors are a bit too strong and dogmatic about the necessity of enhancer-promoter contacts and physical proximity for enhancer function. The authors should acknowledge other mechanisms of action that have been previously proposed (tracking, linking, etc). In this regard, do the authors think that proximal (within a few Kb of a promoter) and distal enhancers control gene expression through similar mechanisms?. For proximal regulatory sequences we don't have many evidences of looping because current 3C-based or imaging methods do not have the resolution to detect such short-range interactions. Perhaps tracking or linking models can explain how proximal enhancers work.

Moreover, recent work from Levine and others, mostly in Drosophila, suggests that distal enhancers might control gene expression through a combination of looping and tracking/linking mechanisms: long-range loops can bring enhancers close to a promoter, but not in direct proximity, and tracking or linking could bridge the distance.

*We do believe that the transcription activating information must come from the enhancer to the promoter through physical contact. The other alternative is diffusible emanation of the regulatory instructions from the enhancer to the promoter, and we do not think this is likely. We do not believe an enhancer can emanate any diffusible instruction to the promoter.*

*We favor the looping model as opposed to linking or tracking. Arguably, the end-point of all the three models (linking, tracking and looping) would be the same direct enhancer-promoter contact, which the existing 3C-based methodologies detect. However, we think it is unlikely for the cells to adopt either linking or tracking mechanisms for all enhancers; because of several reasons. (1) A tracking model will make enhancer action essentially dependent on chromatin motor proteins, such as cohesin or condensin; we have no evidence that these proteins are absolutely essential for enhancer action. (2) The tracking will also be energy- and time-consuming to activate target promoters at the genomic scales. (3) The tracking model would be incompatible with those genes that house intronic enhancers, since tracking would interfere with transcribing RNA polymerases. (4) We do not think linking moieties are possible to link all enhancer-promoter pairs over long distances. But, As the Reviewer raised the possibility, tracking and linking can potentially be a mechanism of action for promoter-proximal enhancer. A similar view was also expressed by Furlong and Levine, 2018 (Furlong and Levine, 2018).*

*To highlight the above, we have added additional text to the manuscript on Page 11.*

- The authors briefly mention at the beginning of the manuscript the main types of cis-regulatory elements, including enhancers and silencers. Later in the text, they state the following: "Proteins assembled at enhancers can promote either activation or repression of target genes depending on their repertoire of coactivator or repressor complexes, respectively." In my opinion, this statement can be quite confusing, since an enhancer that represses its target genes due to the action of co-repressors could define as a silencer.

*Here, we are referring to “inactivation of an enhancer” (via recruitment of corepressor proteins) that could silence a gene. In this context, there are reports that repressive proteins are recruited to enhancers to instigate transcription repression. This is different from “silencer-mediated silencing”.*

In fact, previous as well as recent work in Drosophila that same regulatory elements could work as either enhancers or silencers depending on the proteins bound to them (Gisselbrecht et al., Mol Cell, 2020). Moreover, during the last couple of years there have been a number of papers describing genome-wide identification of silencers, although their mechanisms of action (including whether or not they loop to target promoters) remain quite obscure. I think the authors should have a dedicated section to comment about the differences between enhancers and silencers, maybe in the "Emerging concepts" section.

*We have now added a short sub-section “Enhancer or Silencer?” in the “Emerging Concepts” section on Page 18 that should also alleviate the above confusion.*

- Regarding the "Emerging concepts" section, another topic that I missed in the review was the mechanisms implicated in the control of enhancer-gene compatibility. Accumulating evidences suggest that not all enhancers and genes located within the same TAD or regulatory domain (see previous work and recent preprints from Lenhard, Furlong and Rada-Iglesias laboratories) can physically and functionally communicate with each other. Moreover, there are also emerging evidences of how the genetic composition of both promoters and enhancers as well as the protein complexes recruited to them can control gene-enhancer compatibility (see recent work from Alex Stark lab as well as Pachano et al. bioRxiv, 2020). On the other hand, and as mentioned above, dogmatic statements such as "An enhancer and its cognate target gene always reside within a TAD", since there are some evidences of enhancers acting in trans, including transvection mechanisms in flies. Moreover, TADs might not be a universal of metazoans genomes, while enhancers, as stated by the authors are likely to be so.

*To reflect the reviewer’s concern and suggestion, we have made necessary changes to the TAD paragraph under the section “Controlling enhancer action through chromatin organization”; also included are new context and references on GRB as suggested by Reviewer 2 (Page 17).*

*We also extended the section “Complexity of multi-enhancer control” and revisit this question of enhancer-promoter specificity and selectivity (Page 18).*

- The authors dedicate quite some effort to describe the importance of enhancer-promoter contacts and how this can contribute to enhancer function and transcription. However, I think the authors should better describe the proteins and protein complexes that have been describe so far as regulators of enhancer-gene interactions both in the active as well as poised states.

*We already have a dedicated section for the “proteome of enhancers”. Besides, almost all of these proteins (except a few generic proteins/complexes such as the Mediator) work at certain gene loci, and at present, a generalizable notion of their universal role cannot be derived. Also, except for a few like GATA2 and LDB1, we do not have mechanistic clarity as to how these proteins contribute to enhancer-promoter communications and subsequent transcription. Therefore, even as we welcome and appreciate the reviewer’s suggestion, our understanding on “enhancer proteome” is perhaps still in infancy.*

- Regarding eRNAs and the strong coupling between enhancer and gene transcription, I think the authors should discuss previously proposed models suggesting that both enhancers and gene promoters can act as independent transcriptional units (see work from Andersson and Sandelin lab). Moreover, the authors should also mention previous work suggesting that enhancer and gene transcription are mutually dependent i.e. eRNAs are not produced if gene promoters are eliminated or not compatible (Kim et al, Nature, 2010; Pachano et al, bioRxiv, 2020).

*We are aware of the similar architectures of the enhancers and promoters. We did not focus on this aspect because we were shaping the manuscript as enhancer-centric (though the enhancer-promoter dichotomy is getting somewhat blurry). However, we have added a sentence to reflect this fact (Page 7).*

*Since we are at an advanced stage of a manuscript detailing the mechanism of enhancer-promoter transcriptional interdependence, we chose to not raise this additional topic here.*

Minors comments:

- When mentioning super-enhancers the authors should acknowledge conceptually similar concepts that were previously described and that have been extensively investigated, such as Locus Control Regions (LCRs).

*Yes, we have now added Jason Lieb’s Perspective to the reference (Page 3).*

- Enhancer redundancy might contribute not only to transcriptional robustness but also transcriptional precision.

- "The TFBSs in the majority of enhancers are modular in nature". Do we really have evidences to claim that the majority of TFBS are modular?. I don't think that enough enhancers have been genetically dissected with sufficient spatial resolution to make such a claim. Several models (enhanceosome, billboard, TF collective) should perhaps be briefly presented.

*We have replaced “the majority” with “many”. (Page 5)*

*We have now modified the existing text to introduce the enhancer-proteome models (viz., enhanceosome, billboard, TF collective). (Page 5)*

- "That is, transcription-conducive nucleoprotein architectures at the enhancer and promoter stimulate each other's transcription. The question, then, is what comes first". Is there a need for an order? Could transcription at enhancers and promoters happen simultaneously?. This could be the case if they are mutually dependent rather than if they stimulate each other.

*We think there need not be an order if transcription at the “in contact” enhancer and promoter is interdependent such that something else starts the transcription simultaneously at the enhancer and promoter (but thereafter sustenance of that transcription would be interdependent). We cannot envisage what that something else could be at present.*

*Therefore, we think that for mutually dependent or mutually stimulatory transcription between the enhancer and promoter, one of them has to start transcribing first; so there has to be an order. However, we have no clear idea of this scenario – even as we have recently extensively studied this aspect in vitro (manuscript in preparation). Conceptually, possible determinants of the order could include the position of the RNAPII on the templates within the three-dimensional geometry, and its proximity to CDK7-like kinases that would phosphorylate CTD S5, local availability of NTPs, local geometry of TFiiS etc.*

*To avoid any confusion, we are removing the sentence in question.*

Reviewer #2: The manuscript by Panigrahi and O'Malley presents a review of literature and ideas about enhancers and enhancer-promoter interactions. It is less about enhancers per se and more about what is known about enhancer-promoter complexes (EPCs), which is not a bad thing. I have enjoyed reading it and learned a lot. It is refreshing to read a review written not by one of the "usual suspects" in regulatory genomics, but by researchers developing novel in vitro systems for mechanistic studies of enhancer-promoter interactions. The authors know both the regulatory genomics and mechanistic literature very well. Their choice to put the emphasis on their own angle provides us from the genomics side with a fresh perspective and pointing to many important studies that I suspect many of us will have missed. At the same time, they integrate the discussion of those studies with the results from genomics that we are familiar with.

Given the angle of the manuscript, the title is a bit too broad - "Enhancer action" or "Mechanisms of enhancer action" would be a better match than "Transcriptional enhancers". In the latter case, most Genome Biology readers would expect to find a more conventional treatment of genomic features of enhancers.

*We truly appreciate the reviewer for the kind words, and are happy that we were able to provide a different taste. We also thank the reviewer for suggesting a change to the title. We agree with the suggestion that would make the title more focused. Therefore, we have changed the title to “Mechanisms of enhancer action: The known and the unknown”.*

Specific issues, both major and minor:

- p.3, line 57 "Caspaspora" -> Capsaspora

*We thank the reviewer for catching the error. We corrected it.*

-p.3, "Moreover, microsynteny is evidently conserved across metazoans (ref. 31), indicative of the presence of enhancers in the bystander genes." - the concept of bystander genes vs target genes is part of the Genomic Regulatory Block (GRB) model, introduced in Kikuta H et al. 2007. Genomic regulatory blocks encompass multiple neighboring genes and maintain conserved synteny in vertebrates. 17: 545-555. I think that the GRB model deserves a proper mention in a review about enhancer-promoter interactions, especially since GRBs has since been shown to coincide with the strongest and most deeply conserved TADs devoted to regulation of developmental genes (Harmston N al. 2017. Topologically associating domains are ancient features that coincide with Metazoan clusters of extreme noncoding conservation. Nature communications 8: 441.). The GRBs and corresponding TADs are spanned by clusters of dozens of enhancers, many of which are ultraconserved, and include both the target genes of those enhancers as well as non-responsive bystander genes.

*We fully agree with the reviewer. However, we have kept the section on enhancer evolution as a supplemental text, suggested to appear in a “box”. Thus, to ensure the brevity required by the journal, we are unable to add more descriptive text to this section elaborating microsynteny. However, now we have added Kikuta, 2007 in the reference. (Ref. 34; page 3)*

*We have added a short sentence in the section “Controlling enhancer action through chromatin organization” to incorporate the suggested reference and context (Page 17). For brevity, again, we are unable to elaborate much further.*

- p.4, line 18 "The TEs can navigate genomic loci far and wide" - I don't understand what this is supposed to mean.

*We meant that the transposable elements can hop around in the genome and insert themselves randomly – contributing to regulatory changes. Perhaps our wording was too poetic. We see that this statement is dispensable. So, we have removed it.*

- p.4, line 26 "Genomic duplication events, fractionally or in whole" - Strange wording. Try e.g. "Genomic duplication events, be it tandem duplications of chromosomal regions, duplications of whole chromosomes, or whole genomes". Also, in most cases it is moot which of the two copies is "new".

*We like the reviewer’s suggested wording and have now adopted it (Page 4).*

*Also, we have changed “The newly gained copy” to “One of the copies” (Page 4).*

- p.4, line 53-54 "protein-coding sequences have diverged less compared to the non-coding sequences" - across certain evolutionary distances, this is not entirely true. Within e.g. vertebrate clade, ultraconserved sentences are non-coding sequences that often overlap developmental enhancers and are much more conserved than even the best conserved protein-coding sequences (there are almost 500 such sequences that are identical over 200bp or more between human and mouse, and there are hundreds that are extremely highly conserved between mammals and fish).

*The reviewer is right that there are certain regulatory sequences that are ultraconserved in evolution. But, we are also right in saying that the “protein-coding sequences have diverged less compared to the non-coding sequences” when we take a global, whole-genome perspective. Many genes have remained quite conserved, while their patterns of regulation have changed, and this is a major mechanism behind evolution.*

- p.5, "enhancers such as the human IFNbeta1" - IFNbeta1 is a gene, not an enhancer, you mean an enhancer that regulates IFNbeta1

*Yes. We have reworded it. (Page 6)*

- p.6, line 4: While "evolutive" is a legitimate word, it is not normally used in biology. Use "evolutionary constraints".

*We appreciate the reviewer’s suggestion. We feel that the word “evolutive” is more evocative than ‘evolutionary’. Therefore, we would like to retain it if the reviewer kindly allows.*

- p. 6, line 34-36, and Table 1: Why are reader proteins mentioned as part of the proteome of the enhancers, and writers and erasers are not? E.g. p300 is a writer and is definitely found bound to active enhancers.

*We mentioned them as the “Coregulators”. P300 comes under “chromatin remodeling and modifying complexes” in Table 1. We have now added the terms “writers” and “erasers” for visibility.*

- p. 7, line "the RNAPII and GTFs comprise a sum of 45 proteins" - the exact number and composition depend on the promoter class, cell type, and species.

*The reviewer is right. This number comes from the cited review by Patrick Cramer, and used here as an example for the magnitude of basic minimum proteome of the enhancer/promoter. We have now rephrased the sentence to reflect generality (Page 7):*

*“A typical PIC may comprise the RNAPII and GTFs, totaling up to a sum of 45 proteins with a combined mass of over 2,500 kDa (Sainsbury et al., 2015).”*

- p.7, line 28: "inevitable role" - you probably mean "indispensable".

*We have changed it to "indispensable"*

- p.8, line 14: the idea of "housekeeping enhancers" is still controversial - both in the case of ETS-bound elements (Curina et al.) in mammals and the STARR-seq detected "housekeeping enhancers" in Drosophila (Stark lab), the elements are so close to core promoter that it is equally possible to consider them part of the promoter architecture, and not separate enhancer elements.

*We agree with the reviewer that the current notion of “housekeeping enhancers” is not solid, and that the experimental approach taken here juxtaposes the enhancers to the promoter. However, since there is not much else in the literature on ‘housekeeping enhancers’, we decided to present to the inquisitive reader an opportunity to dig deeper. Therefore, we seek to retain this reference and context.*

-p.8, line 54-56: "Widespread intergenic transcription was detected at genomic scale twenty five years ago and such pervasive transcription was soon mapped to enhancers" - this sounds like enhancer transcription is the main/dominant cause of intergenic transcription. On the contrary, much of the intergenic transcription was due to previously undetected noncoding RNAs (lncRNA and some shorter classes) whose transcription initiation is not thought to universally start at enhancers.

*We agree, and we have now reworded the text to reflect this fact. (Page 9)*

- p.8, line 60 - p.9, line 4: "Studies employing CAGE have estimated that over 43,000 distinct eRNAs can be detected in a human cell" - this is not quite right. First, the study in question (Andersson et al. 2014) defines enhancers as having near-symmetrical bidirectional CAGE signal, so 43,000 enhancers would equal twice as many (86,000) eRNAs. Second, they were not detected in a human cell, but in a cumulative signal from almost 1000 CAGE samples. Third, the number is depth-dependent, and subsequent CAGE experiments detected even more enhancers with bidirectional eRNA signatures.

- Regarding the above, it would be important to mention an alternative explanation of eRNA CAGE signal: see Young RS, Kumar Y, Bickmore WA, Taylor MS. 2017. Bidirectional transcription initiation marks accessible chromatin and is not specific to enhancers. Genome Biol 18: 242.

*We have taken note of the above comments and have edited the paragraph (Page 9).*

- p.9, line 16-17: "lipopolysaccharide- induced eRNA synthesis in mouse macrophages precedes transcription from the target promoter" - while this is the conclusion of Arner et al Science paper, the subsequent NET-CAGE paper reveals it to be a likely artefact of the fact that regular CAGE detects mature RNA, and that eRNA is much less stable than the target promoter mRNA, resulting in faster accumulation of eRNA than mRNA to the steady state levels after simultaneous induction.

*We intended to stay true to the conclusion of Arner et al, but we have mildly edited this paragraph (Page 9).*

- p. 11, line 37: "since the regulatory control of gene expression in higher eukaryotes is essentially uncoupled from the act of target gene transcription per se, it is imperative that these two steps are connected" - I am not sure what this means; it reads like "since X and Y are uncoupled, it is essential that they get coupled". But if they get coupled then they are not uncoupled. Please rephrase.

*By “uncoupled”, we meant the vast distance between the promoter and regulatory sequences in higher eukaryotes, compared to the gene-proximal regulatory sequences in prokaryotes and early unicellular eukaryotes. We have rephrased it. Heeding Reviewer 1, we have restructured this portion to include a brief account of other models of enhancer-promoter communication. (Page 11-12)*

- p.16, line 32-34: "TADs are demarcated by CTCF-bound insulator elements" - that is not universally true for all Metazoa (e.g. Drosophila).

*We have modified the statement to include “in vertebrates” (Page 17).*

- p. 17, line 4: "Cohesion-mediated" -> "cohesin-mediated"

*We thank the reviewer for catching the error; we have now fixed it.*

- p. 17, line 30-32: "According to a new report, the most distal enhancer in an enhancer chain usually acts as the predominant regulator of a target gene (ref 236)" - Based on what ref. 236 actually shows and how it shows it, I would not make such a strong claim out of it.

*While we agree that the claim is not strong, we decided to leave it to the curious reader to evaluate the author’s claims and develop more informed understanding. Our wording “According to a recent report” also testifies that more evidence is required here.*

- p.17, line 51: "a multi-modular structure called 'multivalency' " - please cite reference where this structure is first suggested (it is not clear at the moment).

*We have added the reference (Li et al., 2012); we have also made a minor modification to the sentence (Page 19).*

- p. 18, line 41: "Such direct contacts between the chromatin-anchored transcripts is possible" -> "... ARE possible"

*Thank you; we have corrected it.*

*Furlong, E.E.M., and Levine, M. (2018). Developmental enhancers and chromosome topology. Science 361, 1341-1345.*

*Li, P., Banjade, S., Cheng, H.C., Kim, S., Chen, B., Guo, L., Llaguno, M., Hollingsworth, J.V., King, D.S., Banani, S.F., et al. (2012). Phase transitions in the assembly of multivalent signalling proteins. Nature 483, 336-340.*

*Sainsbury, S., Bernecky, C., and Cramer, P. (2015). Structural basis of transcription initiation by RNA polymerase II. Nat Rev Mol Cell Biol 16, 129-143.*
